# *Lactobacillus plantarum* Generate Electricity through Flavin Mononucleotide-Mediated Extracellular Electron Transfer to Upregulate Epithelial Type I Collagen Expression and Thereby Promote Microbial Adhesion to Intestine

**DOI:** 10.3390/biomedicines11030677

**Published:** 2023-02-23

**Authors:** Binderiya Ganzorig, Enkhbat Zayabaatar, Minh Tan Pham, Shinta Marito, Chun-Ming Huang, Yu-Hsiang Lee

**Affiliations:** 1 Department of Biomedical Sciences and Engineering, National Central University, Taoyuan City 320317, Taiwan; 2 Faculty of Applied Sciences, Ton Duc Thang University, Ho Chi Minh City 700000, Vietnam; 3 Department of Chemical and Materials Engineering, National Central University, Taoyuan City 320317, Taiwan

**Keywords:** *L. plantarum*, electricity, carbon source, flavin mononucleotide, extracellular electron transfer, microbial adhesion, intestine, type I collagen

## Abstract

The mechanism behind how flavin mononucleotide (FMN)-producing bacteria attach to a host intestine remains unclear. In order to address this issue, this study isolated the Gram-positive bacteria *Lactobacillus plantarum* from Mongolian fermented Airag, named *L*. *plantarum* MA. These bacteria were further employed as the model microbes, and their electrogenic properties were first identified by their significant expression of type II NADH-quinone oxidoreductase. This study also demonstrated that the electrical activity of *L. plantarum* MA can be conducted through flavin mononucleotide (FMN)-based extracellular electron transfer, which is highly dependent on the presence of a carbon source in the medium. Our data show that approximately 15 µM of FMN, one of the key electron donors for the generation of electricity, can be produced from *L. plantarum* MA, as they were cultured in the presence of lactulose for 24 h. We further demonstrated that the electrical activity of *L. plantarum* MA can promote microbial adhesion and can thus enhance the colonization effectiveness of Caco-2 cells and mouse cecum. Such enhanced adhesiveness was attributed to the increased expression of type I collagens in the intestinal epithelium after treatment with *L. plantarum* MA. This study reveals the mechanism behind the electrogenic activity of *L. plantarum* MA and shows how the bacteria utilize electricity to modulate the protein expression of gut tissue for an enhanced adhesion process.

## 1. Introduction

*Lactobacillus plantarum* (*L*. *plantarum*) is a facultative Gram-positive lactic acid bacterium that has been used extensively in the dairy industries for its ameliorative properties to the intestinal environment, as well as its benefits to gastroenteric health [1]. Microorganisms such as *L*. *plantarum*, which are extracted from Mongolian mare milk-drink (Airag), may also provide increased antioxidant activity when compared to other Lactobacilli strains, as previously reported [2]. *L. plantarum* has eight-gene locus codes which are utilized for nicotinamide adenine dinucleotide (NADH) dehydrogenase and can generate electricity through extracellular electron transfer (EET); such electrical activity is highly correlated to the level of NAD^+^ in the surrounding environment [3]. Microbial electrical production using EET is a key survival feature of electrogenic bacteria in the gut environment, however, the host–microbe interaction remains unclear [4].

EET allows electrons to be transported from the microbial cytosol to external conductors such as electrodes and/or minerals, and plays an important role in bacterial physiology including growth [3], colonization [5], and fermentation [6]. For example, it has been reported that both *Bacillus cereus* DIF1 and *Rhodococcus ruber* DIF2 can secrete redox mediators such as riboflavin and/or flavin mononucleotide (FMN) to promote their growth [7]. Electricity generated from *Clostridium cochlearium* in mice guts can enhance bacterial growth and fermentation [6]. Our previous work demonstrated that electricity produced from the Gram-positive lactic acid bacteria *Leuconostoc mesenteroides* is favorable for bacterial colonization in the intestine [5]. More electrogenic Gram-positive bacteria such as *Enterococcus faecalis*, *Faecalibacterium prausnitzii* and *Lactococcus lactis* which enable flavin-mediated EET were recently discovered by use of a microbial fuel cell chamber (MFCC) [8,9,10].

Colonization of probiotics such as *L*. *plantarum* can trigger the immune system and eliminate pathogenic bacteria [11]. However, *L*. *plantarum* does not naturally exist in the human body and must be taken exogenously through dietary means [12]. Bacterial adhesion to the human gut is typically carried out through binding with the extracellular matrix (ECM) proteins such as collagen, fibronectin, and/or laminin of the epithelium at the mucus layer [13], while collagen type I (Col-I) is the most prevalent type of ECM protein in the intestinal tissues [14]. Binding with collagen is often the first step for microbes to be colonized on the sub-intestinal mucus [15], and it has been demonstrated that 75% of *Lactobacillus* isolated from humans and/or animals achieve intestinal adhesion through the use of collagen-binding proteins located on the surface of the bacteria [16]. There are two types of collagen-binding adhesin proteins on the *Lactobacillus* surface, named the ATP-binding cassette (ABC) transporter system and the S-layer proteins [17,18]. The Col-I binding protein is one of the ABC proteins on the *Lactobacillus* surface that has a high affinity for Col-I on the intestinal mucosa [19], while expression of the S-layer protein can be modulated via the level of collagen in the colon [20]. Such circumstances indicate that the level of Col-I expression in the intestine plays a crucial role in the colonization of *L. plantarum* in the gastrointestinal tract. However, the mechanism by which electrogenic bacteria (e.g., *L. plantarum*) affects collagen expression of intestinal cells needs further elucidation.

In this paper, we aim to investigate the correlation between electricity and the adhesion capability of *L. plantarum* MA to the intestine, and how microbial electricity is affected by the culture environment. Lactulose, a disaccharide composed of galactose and fructose, is a selective carbon source (prebiotic) for *L. plantarum* fermentation [21] and can stimulate the growth of Gram-positive bacteria, since it may increase the quantity of short-chain fatty acids (SCFA) including acetic, butyric, and lactic acids [22] and may therefore improve the microflora environment in vivo [23]. In this study, *L. plantarum* extracted from Mongolian Airag were investigated for their electrical properties and colonization capabilities in the presence and absence of lactulose in vitro and ex vivo.

## 2. Materials and Methods

### 2.1. Bacterial Identification and Cultivation

The *L. plantarum* that were utilized in this study were self-isolated from Mongolian fermented Airag (named *L. plantarum* MA) and identified/confirmed using the 16S ribosomal RNA sequencing method (BigDye Terminator V3.1 Cycle Sequencing Kit, ThermoFisher Scientific, Waltham, MA, USA). Both *L. plantarum* MA and *Lactobacillus pentosus* (*L. pentosus*; ATCC^®^ 8041, ATCC, Rockville, MD, USA) were regularly cultured in shaken de Man-Rogosa-Sharpe broth (MRS, Hi-Media, Mumbai, India) under aerobic conditions at 37 °C. *Staphylococcus epidermidis* (*S. epidermidis*; ATCC^®^ 12228, ATCC) were cultivated in shaken tryptic soy broth (TSB, Sigma, St. Louis, MO, USA) under aerobic conditions at 37 °C. The microbes were cultured with 2% of lactulose or glucose according to the experimental setup. The number of bacteria was evaluated using spectrophotometry at λ_abs_ = 600 nm. Sub-cultivation of the bacteria was performed using 1:100 dilution, when the value of the optical density (OD_600_) was ≥1.0.

### 2.2. Quantification of Microbial NDH-2 Expression by RT-qPCR

The expression levels of the type II NADH-quinone oxidoreductase (NDH-2) gene in *L*. *plantarum* MA, *L*. *pentosus*, and *S*. *epidermidis* were quantitatively measured using a RT-qPCR. *L. pentosus* is the known electrogenic bacteria which can produce electricity without mediators [24] and therefore served as the positive control in this assay. In brief, bacterial RNA from 1 × 10^8^ microbes was extracted using the RNA Mini-Purification Kit (Biokit Biotechnology Inc., Miaoli, Taiwan) followed by DNase treatment to remove DNA contamination, according to the manufacturer’s instructions. The purity of the isolated RNA was assessed via spectrophotometry, where the accepted range of A260/A280 was between 1.8–2.0. Afterward, the total RNA was converted to cDNA using the iScript cDNA Synthesis Kit (Bio-Rad, Hercules, CA, USA) and the products were immediately subjected to qPCR or stored at −20 °C until use.

SYBR Green qPCR was performed to quantitatively detect the expression level of the NDH-2 transcripts in each microbial group. The qPCR sample was prepared by combining 2× qPCR Master Mix (Thermo Fisher Scientific, Waltham, MA, USA), primers (0.4 µM; Table 1), and 2 µL of the cDNA template. The total volume of each reaction was adjusted to 10 µL using nuclease-free water. The qPCR associated with the melting-curve analysis was carried out using the StepOnePlus RT PCR System (Thermo Fisher Scientific) which was incorporated with a fixed thermal-cycling program set at 95 °C for 10 min, 95 °C for 15 s, 60 °C for 60 s, and 72 °C for 30 s, leading to a total of 40 cycles. All primers (Table 1) used in RT-qPCR were designed with an intron-spanning assay through a Primer-Blast tool (https://blast.ncbi.nlm.nih.gov/Blast.cgi/ (accessed on 20 December 2022)) and produced by Sigma. Gene expression was determined using the cycle threshold (2^−ΔΔCt^) and was quantitatively analyzed against the blank after normalization.

### 2.3. Detection of Microbial Electricity In Vitro

**Figure 1:**
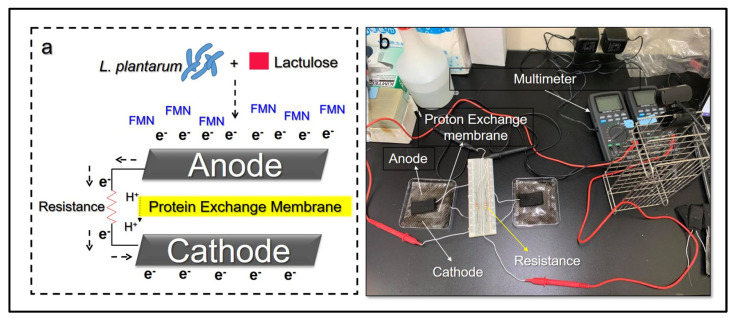
Identification of the bacteria isolated from the Mongolian fermented Airag. (**a**) Schematic diagram of the MFCC operations. (**b**) Photograph of the real MFCC device.

Electricity generated from the microbials was detected using a microbial fuel cell chamber (MFCC) assembled according to a previous study [25]. In brief, the MFCC was fabricated using carbon cloth (Homy Tech, Taoyuan, Taiwan) and carbon felt (Homy Tech) for the cathode and anode, respectively, with 1000 Ω of electrical impedance placed between the two electrodes. In addition, a Nafion^TM^ membrane N117 (Chemours, Wilmington, DE, USA) was set in the middle of the chamber to serve as a proton exchange membrane (PEM). The whole MFCC instrumental setup, along with illustrations, are shown in Figure 1a,b, respectively. To measure bacterial electricity, 10^7^ colony forming units (CFU) per mL of *L. plantarum* MA or *L. pentosus* were first incubated in 5 mL of MRS broth in the presence or absence of a carbon source (i.e., 2% of lactulose or glucose) at 37 °C for 24 h. After transferring to the MFCC, the voltage generated from each group was detected continuously for 20 min using a digital multimeter (Lutron, DM-9962SD, Sydney, Australia).

### 2.4. Detection of Endogenous FMN Yielded from L. plantarum MA

*L. plantarum* MA (10^7^ CFU/mL) pretreated with 1 µM of roseoflavin, which is a FMN inhibitor, or dimethyl sulfoxide (DMSO) were separately incubated for 24 h in 10 mL of MRS broth, in the presence or absence of 2% lactulose. Afterward, the bacteria were centrifuged at 6000 rpm for 15 min and the supernatants were collected in stages, filtrated using 0.22-μm filters, and stored at 4 °C. The quantity of FMN in each supernatant was analyzed using a high-performance liquid chromatography (HPLC) (Alliance-HPLC e2695 System, Waters Corp. Milford, MA, USA) equipped with a ZORBAX Eclipse XDB-C18 column (250 mm × 4.6 mm ID, 5-μm particle size; Agilent Technologies, Santa Clara, CA, USA). Each supernatant was initially dissolved in 1 mL of HPLC-grade methanol and separated in a mobile phase consisting of 20 mM of ammonium acetate (pH 5.4) and methanol at 25 ± 2 °C under 1 mL/min of flow rate. The concentration of methanol in the mobile phase was first set at 5% in the initial isocratic step for 6 min, then gradually increased to 34.5% for 6 min, 37% for 16 min, and 95% for 10 min, followed by a rapid drop to 5% for 16 min. The eluted signal was measured using the spectrophotometry set at λ = 275 nm and evaluated for FMN quantification using the ChromQuest v3.1.6 System (Thermo Fisher Scientific) in association with OriginPro (2022 SR1, OriginLab, Northampton, MA, USA).

### 2.5. Cell Culture

The Caco-2 cells (ATCC^®^ HTB-37™, ATCC) were cultured with Dulbecco’s Modified Eagle medium (DMEM) supplemented with 20% fetal bovine serum (FBS) and 100-U/mL penicillin/streptomycin at 37 °C with 5% CO_2_.

### 2.6. Microbial Adhesion Assay In Vitro

Caco-2 cells with 10^5^ cells/well in 96-well plates were initially washed 3 times with PBS before the experiment. Next, 200 µL of cell culture medium without antibiotics, containing 10^7^ CFU/mL of *L. plantarum* MA and designated agents, were added to the Caco-2 cells, as described in Table 2. After co-incubation with the cells for 60 min, the cells were trypsinized and added to 25 µL of hypotonic tissue protein extraction reagent (T-PER) for the collection of adhered bacteria. To quantify the adhesion capability of *L. plantarum* MA with different treatments, 10 µL of the collected *L. plantarum* MA was seeded on the MRS agar plates using different dilution factors. After maintenance at 37 °C for 12 h, the in vitro adhesion capability of each group was determined based on the value of the microbial population index (MPI) defined by the logarithm of the CFU (MPI = Log_10_ ((CFU + 1)/mL)), as previously reported [5].

### 2.7. Ethics Statement for the Animal Study

A total of 12 Institute of Cancer Research (ICR) mice (8–9 weeks, female) weighing between 25–35 g were employed to build the animal models in this study. All the animal procedures, including the care and operations of the experimental mice, were in accordance with the guidelines approved by the Institutional Animal Care and Use Committee (IACUC) at National Central University (Approval number: NCU-106-016. Approval Date: 19 December 2017).

### 2.8. Microbial Adhesion Assay Ex Vivo

To evaluate the adhesion capability of *L. plantarum* MA on the intestine in vivo, an ex vivo microbial adhesion assay was conducted on murine cecal tissue, since cecum is the main site for bacterial fermentation and electron production [26]. After the mice were anesthetized using CO_2_, the cecum from all mice were collected, washed with 0.9% NaCl to remove the feces, and cut into pieces of approximately equal weight. Afterward, the cecum pieces were separately incubated with *L. plantarum* MA under various conditions, as described in Table 3. After maintenance at 37 °C for 20 h, each cecum sample was washed 3 time with PBS and incubated with 50-µL T-PER and 3-µL protease inhibitor at 37 °C for 10 min, followed by mashing the cecum with a pestle. In this study, the ex vivo adhesion capability of each group was determined based on the MPI, which was assessed by seeding 10 µL of various dilutions of the homogenized samples on MRS agar plates, as described above.

### 2.9. Measurement of Col-I Expression of the Cecum after Various Treatments Ex Vivo

The expression level of Col-I of each cecum sample after microbial treatment was evaluated using a Western blot. Briefly, after various microbial treatments, the homogenized cecum were centrifuged using 15,000 rpm at 4 °C for 10 min and the supernatants were subjected to a bicinchoninic acid assay (BCA) to quantify the total protein that was extracted. Next, 20 µg of protein from each cecum was mixed with a 4× Laemmli sample buffer (Bio-Rad, Hercules, CA, USA) and immediately separated via electrophoresis in 10% sodium dodecyl sulfate polyacrylamide gels (SDS-PAGE) using 100 V of electricity. The gels were then blotted onto a polyvinylidene difluoride membrane (Millipore, Temecula, CA, USA) and incubated with primary antibodies of Col-I (1:2000, Cusabio Technology, Houston, TX, USA) or β-actin (1:5000, Cusabio Technology) for 2.5 h, followed by conjugation with the secondary antibody at a dilution of 1:2000 for 60 min. After washing 3 times with PBS-Tween-20 (0.05% (*v*/*v*); PBS-T), the membranes were treated with chemiluminescent substrates for 5 min and the level of each Col-I or β-actin band was visualized using the Omega Lum™ C Imaging System (Gel company, San Francisco, CA, USA). The β-actin was employed as the reference gene in all Western blot analyses. The blot intensity of each Col-I band was quantified using the ImageJ software and analyzed after normalization against that of the β-actin.

### 2.10. Statistical Analysis

All data were acquired from ≥ three independent experiments and are presented as the mean ± standard deviation (SD). Statistical analyses were conducted using the GraphPad Prism software, in which comparisons between groups were performed using the Student’s *t*-test and 2-way ANOVA, with a significance level of * *p* < 0.05 throughout the study.

## 3. Results and Discussion

### 3.1. Identification and Electrogenic Properties of L. plantarum MA

Supplementary Materials S1 show the 16S rRNA gene sequence of the microbials isolated from the Mongolian Airag (i.e., *L. plantarum* MA). The result shows that *L. plantarum* MA shares 99.6% of its genes with the *L. plantarum* KLDS 1.0728 strain, demonstrating that it is a strain of *L. plantarum*.

**Figure 2:**
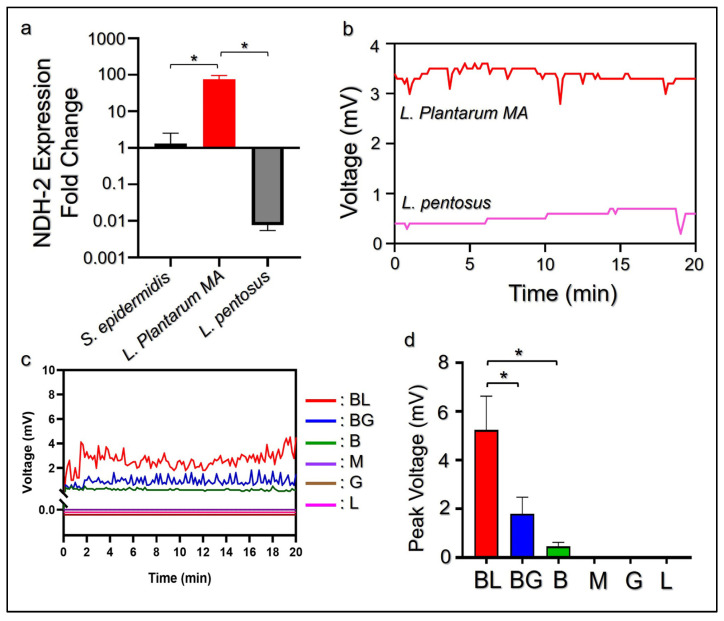
Assessment of the electrogenic property of *L. plantarum* MA. (**a**) NDH-2 expressions of the *S. epidermidis*, *L. plantarum* MA and *L. pentosus* represented by fold change. (**b**) The pattern of electricity production of *L. plantarum* MA and *L. pentosus* which were treated with 2% lactulose within 20 min. (**c**) The pattern of electricity production generated from the MRS (B), MRS + 2% glucose (G), and MRS + 2% lactulose (L), or that were generated from *L. plantarum* MA treated with MRS (B), MRS + 2% lactulose (BL), or MRS + 2% glucose (BG) within 20 min. (**d**) The average peak voltage of each group shown in (**c**). Values are the mean ± SD (n = 3). * *p* < 0.05.

To investigate the electrogenic characteristics of *L. plantarum* MA, the gene expression of NDH-2, one of the key proteins in the EET mechanism [3], was examined using a RT-qPCR assay. As shown in Figure 2a, *L. plantarum* MA exhibited significantly higher levels of NDH-2 compared with that gained from *S. epidermidis* or *L. pentosus* (*p* < 0.05 for each). Furthermore, through the detection of electricity using MFCC, we found that *L. plantarum* MA in the presence of lactulose were able to generate >3-mV of electricity within 20 min, while *L*. *pentosus* (a known electrogenic bacteria) produced <1 mV over the same time period (Figure 2b). These data clearly demonstrate that *L. plantarum* MA is an electrogenic bacteria and that is consistent with the known electrical properties of *L. plantarum*, as previously reported [3].

To further investigate how carbon source affects the production of electricity of *L. plantarum* MA, the microbes cultured with lactulose or glucose were placed separately in the MFCC and an in situ voltage detection was performed for 20 min. As shown in Figure 2c, no voltage change was observed in the groups without bacteria (Figure 2c, M, G, and L), while a tiny voltage fluctuation in the range of 0.05–0.31 mV was detected in the group of *L. plantarum* MA without a carbon source (Figure 2c, B). However, electricity production of *L. plantarum* MA was markedly enhanced in the presence of a carbon source such as lactulose (Figure 2c, BL) or glucose (Figure 2c, BG), where the peak voltage obtained in the former was approximately 2.8 folds (Figure 2d, *p* < 0.05) higher than that of the latter within 20-min detection. These results clearly show that the electrogenic activity of *L. plantarum* MA is highly dependent on the presence of a carbon source, and the lactulose may induce a higher electricity yield compared to glucose with an equal concentration.

In fact, lactulose is a common selective carbon source for *L. plantarum* fermentation [21] and its fermentative byproducts, including acetic acid and FMN, can serve as electron donors for the generation of electricity, as previously reported [27]. Since *L. plantarum* with lactulose can produce a higher amount of acetic acid in fermentation compared to that with glucose [28], we reason that the higher electricity led by lactulose, as opposed to glucose, is attributable to increased fermentation byproducts in the medium.

### 3.2. Effect of FMN on Electricity Generation of L. plantarum MA

**Figure 3:**
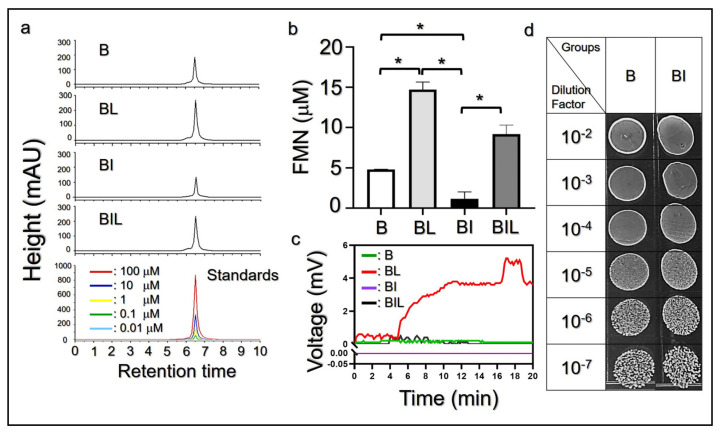
Effect of roseoflavin on the FMN production of *L. plantarum* MA. (**a**) HPLC chromatograms of FMN produced from *L. plantarum* MA treated with 2% lactulose (BL), 1-µM roseoflavin (BI), or 1-µM roseoflavin + 2% lactulose (BIL). Bacteria treated with normal MRS broth (B) was employed as the control. The chromatograms of the standard FMN in various concentrations exhibit that the FMN peak is appeared at approximately the 6.5th min. (**b**) Quantification of the FMN of each group shown in (**a**). Values are the mean ± SD (n = 3). * *p* < 0.05. (**c**) Patterns of electricity productions of *L. plantarum* MA under treatment of nothing (i.e., MRS only; B), lactulose (BL), roseoflavin (BI), or roseoflavin + lactulose (BIL) for 20 min. (**d**) Photographic images of the colonies of *L. plantarum* MA which were pretreated with nothing (B) or roseoflavin (BI) for 24 h before being subjected to the colony assay. Images in the six rows represent the colony forming conditions made by seeding 10^2^–10^7^-fold bacterial dilutions. All images were photographed using a digital camera after cultivation on the MRS agar plates for 24 h.

We further investigated how the fermentation product FMN was influenced by lactulose. Figure 3a shows the HPLC chromatograms of FMN fermented from *L. plantarum* MA with various treatments. Based on the spectrophotometric analysis, *L. plantarum* MA with lactulose generated significantly enhanced FMN compared to the bacteria without a carbon source (Figure 3b, B vs. BL, *p* < 0.05); the latter was dramatically decreased by ~80% after treatment with roseoflavin for 24 h (Figure 3b, B vs. BI, *p* < 0.05). However, roseoflavin-mediated FMN impairment in *L. plantarum* MA can be effectively mitigated by the addition of lactulose, as plotted in Figure 3b. The bioelectrical productivity of the bacterial culture media with the above settings and the subsequent results are presented in Figure 3c. Our data show that the intensity of voltage is ranked by the group + lactulose (Figure 3c, BL) >> + (roseoflavin + lactulose) (Figure 3c, BIL) > no treatment (Figure 3c, B), while the electricity of the group with roseoflavin (Figure 3c, BI) was shown to be at 0 mV throughout the 20-min detection sequence. This outcome is positively correlated with the data of FMN production, as plotted in Figure 3b. On the other hand, FMN expression is independent of the microbial viability and/or growth, since *L. plantarum* MA with and without roseoflavin displayed similar colony patterns, as shown in Figure 3d.

Pretreatment with a riboflavin kinase inhibitor (i.e., roseoflavin) can reduce the electricity production in Gram-positive bacteria [29]. In this study, we showed that a 24 h pretreatment of *L. plantarum* MA with roseoflavin significantly reduced the concentration of FMN by approximately 12 folds (Figure 3b) while also decreasing electricity production (Figure 3c), suggesting that the bioelectrical activity of *L. plantarum* MA was mediated through the FMN-based EET system. On the other hand, it was reported that roseoflavin may target a FMN riboswitch and impair the bacterial growth under concentrations of 100 µM [30]. In this study, roseoflavin was employed with only 1 µM and thus no detrimental effect was found to the growth of *L. plantarum* MA (Figure 3d). Taken together, these results clearly demonstrate that a carbon source (e.g., lactulose) plays a crucial role in the bioelectrical properties of *L. plantarum* MA since it can modulate the production of FMN, which is one of the key electron mediators for EET activity [13].

### 3.3. Effects of FMN-Mediated Electricity on Microbial Adhesion In Vitro and Ex Vivo

**Figure 4:**
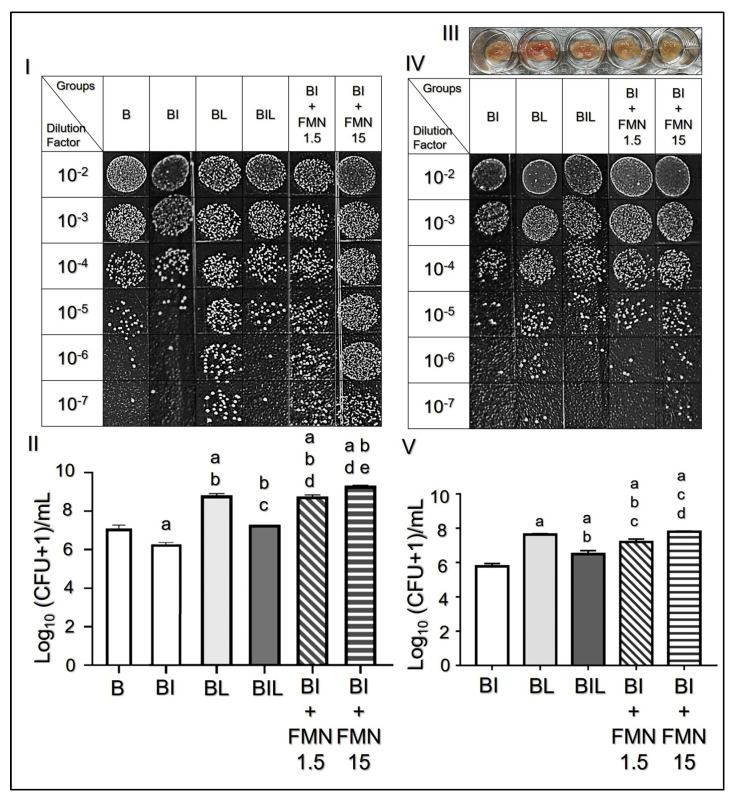
Evaluation of the adhesion capability of *L. plantarum* MA to the intestine in vitro and ex vivo. (**I**) Photographic images of the colonies of cell-attached *L. plantarum* MA grown on the MRS agar plates for 24 h. The bacteria were co-cultured with the Caco-2 cells in the presence of nothing (B), roseoflavin (BI), lactulose (BL), roseoflavin + lactulose (BIL), roseoflavin + 1.5 µM FMN (BI + FMN 1.5), or roseoflavin + 15 µM FMN (BI + FMN 15) for 24 h, followed by isolation from the cells for the colony assay. (**II**) Quantitative analyses of the MPI of *L. plantarum* MA shown in (**I**). Values are the mean ± SD (n = 3). a, b, c, d, and e represent *p* < 0.05 compared to the groups treated with B, BI, BL, BIL, and BI + FMN 1.5, respectively. (**III**) Photographs of the cecum used in the adhesion assay ex vivo. (**IV**) Photographic images of the colonies of the cecum-attached *L. plantarum* MA grown on the MRS agar plates for 24 h. *L. plantarum* MA were co-cultured with cecum in the presence of roseoflavin (BI), lactulose (BL), roseoflavin + lactulose (BIL), roseoflavin + 1.5 µM FMN (BI + FMN 1.5), or roseoflavin + 15 µM FMN (BI + FMN 15) for 24 h, followed by isolation form the cecum for the colony assay. (**V**) Quantitative analyses of the MPI of *L. plantarum* MA shown in (**IV**). Values are the mean ± SD (n = 3). a, b, c, and d represent *p* < 0.05 compared to the groups treated with BI, BL, BIL, and BI + FMN 1.5, respectively.

To evaluate how electricity influences the interactions between *L. plantarum* MA and human intestines, the adhesion efficacies of the conditional *L. plantarum* MA to the intestine in vitro and ex vivo were examined step-by-step. As shown in Figure 4I,II, it can be observed that *L. plantarum* MA with lactulose exhibited a significantly enhanced adhesiveness to Caco-2 cells, while the *L. plantarum* MA with roseoflavin showed an opposite result compared to the setting without a carbon source (Figure 4II, *p* < 0.05). However, the roseoflavin-led reduction in cell adhesion can be ameliorated by simultaneously treating the bacteria with lactulose or exogenous FMN, as displayed in Figure 4I. Based on the CFU analysis, the MPI of the group with roseoflavin + FMN was 43% and 55% (Figure 4II, *p* < 0.05 for each) higher than the one with roseoflavin alone when the dose of FMN was set as 1.5 and 15 µM, respectively. These outcomes indicate that an electron mediator (i.e., FMN) does play a crucial role in the gut adhesion of *L. plantarum* MA, and its production is highly dependent on the supply of carbon source (e.g., lactulose) in the medium (Figure 3b).

Similar results can be found in the ex vivo assay (Figure 4III), where lactulose as well as FMN, but not roseoflavin, facilitated microbial adhesion to mouse cecum (Figure 4IV). Based on the quantitative analysis of the CFUs, the MPI of the group concurrently treated with roseoflavin + lactulose, roseoflavin + 1.5 µM of FMN, or roseoflavin + 15 µM of FMN was 10%, 24%, and 35% higher than the one with roseoflavin alone (Figure 4V, *p* < 0.05 for each), implying that the presence and concentration of FMN is pivotal for the adhesion of *L. plantarum* MA in vivo. Taken together, we reason that lactulose/FMN-enhanced bacterial adhesion to the intestine results from the enhanced electricity of *L. plantarum* MA, since lactulose may facilitate the production of electron mediators (e.g., FMN) and increase microbial bioelectric activity [4]. On the contrary, roseoflavin inhibited FMN production and thus diminished microbial electricity, resulting in reduced bacterial colorization in the gut.

### 3.4. FMN Upregulates Col-I Collagen Expression on Cecum

**Figure 5:**
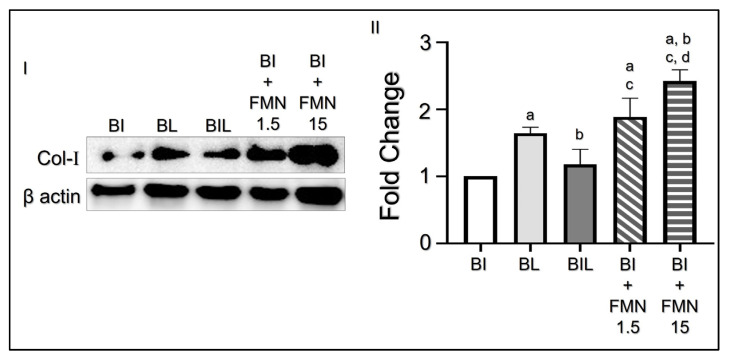
Col-I expressions of the cecum after different treatments. (**I**) Western blot images of Col-I and β-actin of each mouse cecum after treatment with *L. plantarum* MA in the presence of roseoflavin (BI), lactulose (BL), roseoflavin + lactulose (BIL), roseoflavin + 1.5 µM FMN (BI + FMN 1.5), or roseoflavin + 15 µM FMN (BI + FMN 15) for 24 h. (**II**) Quantitative analyses of the Col-I expressions shown in (**I**). Values are the mean ± SD (n = 3). a, b, c, and d represent *p* < 0.05 compared to the groups treated with BI, BL, BIL, and BI + FMN 1.5, respectively.

To verify the rationale for the proposed hypothesis of enhanced microbial adhesion, we further examined how the mouse cecum responded to FMN/electricity using a Western blot. As shown in Figure 5I, it can be observed that the group with *L. plantarum* MA and lactulose exhibited a remarkably enhanced Col-I expression compared with that gained from the *L. plantarum* MA + roseoflavin. Furthermore, addition of lactulose or FMN was indeed able to restore the Col-I expression level suppressed by roseoflavin. Based on the quantitative analyses of the blot intensities (Figure 5II), the expression level of Col-I of the cecum treated with roseoflavin + lactulose, roseoflavin + 1.5 µM of FMN, or roseoflavin + 15 µM of FMN was 14.6%, 28% (*p* < 0.05), and 40.7% (*p* < 0.05) higher than the one with roseoflavin alone. Taken together with the results of FMN-mediated EET activity shown in Figure 3c, these outcomes indicate that the Col-I expression of the intestinal tissue can be upregulated using microbial electricity and hence can promote the bacterial adhesion to the gut epithelial cells ex vivo.

It has been widely demonstrated that electron mediators/currents play an important role in bacterial aggregation, colonization, and motility [31]. In this study, we demonstrated that the addition of either lactulose or FMN to the roseoflavin-pretreated *L. plantarum* MA was indeed able to promote adhesiveness of the bacteria in vitro and ex vivo (Figure 5), and was also able to dramatically enhance the Col-I expression of the attached cecum, as illustrated in Figure 5I and Figure 5II, respectively. However, direct treatment with FMN may provide significantly higher microbial adhesion and Col-I expression compared to bacteria treated with lactulose, suggesting that the FMN-mediated EET is the central mechanism for modulation of their colonization, where *L. plantarum* MA can swiftly utilize FMN to generate electricity and upregulate collagen expression of the gut tissue for an enhanced microbial adhesion. Nonetheless, further investigation into how molecular signaling of the intestine correlates with microbial electricity is certainly needed and efforts are currently in progress.

## 4. Conclusions

In this study, we have successfully isolated *L plantarum* from Mongolian fermented Airag, named *L. plantarum* MA, and identified their electrogenic property due to significant NDH-2 expression. We demonstrated that a carbon source such as lactulose plays a crucial role in the generation of microbial electricity for *L. plantarum* MA, where the produced FMN can serve as electron mediators for the activity of extracellular electron transfer. Furthermore, we demonstrated that *L. plantarum* MA can upregulate the Col-I expression of the intestinal cells as well as tissues in the presence of lactulose or FMN, and thereby enhance the bacterial adhesion in vitro and ex vivo. Since *L. plantarum* are a type of probiotic which can enhance nutrient digestibility, modulate immune response, and eliminate pathogenic microbes [32], the efforts presented in this study may provide a new insight into the dietary applications of *L. plantarum*-containing food such as Mongolian fermented Airag.

## Figures and Tables

**Table 1:** Primers of the NDH-2 transcripts for three microbial sets.

Microbial Species	Primers (F: Forward; R: Reverse)
*L. plantarum* MA	F: AACACGCCATCAACTGGATGT R: TTTAGTCCAGCAGTCCCATAACC
*L. pentosus*	F: GCAGAACTCGACCCTGCAAA R: TGCGTCCGATTGAGCATGT
*S. epidermidis*	F: TGGACAGCAGGCATACAACC R: TCTGCTAGTTGAGCACTGGG

**Table 2:** Experimental design for microbial adhesion assay in vitro.

Microbial Species	Adhesion Target	Treatment (Dose)
*L. plantarum* MA	Caco-2 Cells	* None
		Glucose (2% *w*/*w*)
		Lactulose (2% *w*/*w*)
		Roseoflavin (1 µM)
		Roseoflavin (1 µM) + Lactulose (2% *w*/*w*)
		Roseoflavin (1 µM) + 1.5 µM FMN
		Roseoflavin (1 µM) + 15 µM FMN

* The Caco-2 cells and *L. plantarum* MA were co-cultured in regular cell growth medium without antibiotics.

**Table 3:** Experimental design for microbial adhesion assay ex vivo.

Microbial Species	Adhesion Target	Treatment (Dose)
*L. plantarum* MA	Mice cecum	Roseoflavin (1 µM)
		Lactulose (2% *w*/*w*)
		Roseoflavin (1 µM) + Lactulose (2% *w*/*w*)
		Roseoflavin (1 µM) + 1.5 µM FMN
		Roseoflavin (1 µM) + 15 µM FMN

## Data Availability

The datasets created and/or analyzed in this work are available upon request.

## Supplementary Materials

The following supporting information can be downloaded at: https://www.mdpi.com/article/10.3390/biomedicines11030677/s1, S1: Result of 16sRNA sequence of *L. plantarum* MA.
