# Supplementary material for: Lactobacillus plantarum Generate Electricity through Flavin Mononucleotide-Mediated Extracellular Electron Transfer to Upregulate Epithelial Type I Collagen Expression and Thereby Promote Microbial Adhesion to Intestine
Source: Biomedicines. 2023 Feb 23;11(3):677. doi: 10.3390/biomedicines11030677 (PMC10045142; doi:10.3390/biomedicines11030677)
Supplement: Supplementary file 1 [file biomedicines-11-00677-s001.zip › biomedicines-2171154-supplementary.pdf]

Lactobacillus plantarum Generate Electricity Through Flavin Mononucleotide-Mediated Extracellular Electron Transfer to Upregulate Epithelial Type I Collagen Expression and thereby Promote Microbial Adhesion to Intestine

Binderiya Ganzorig[1], Enkhbat Zayabaatar[1], Minh Tan Pham[2], Shinta Marito[1], Chun-Ming Huang[1], Yu-Hsiang Lee[1,3,*]

[1] Department of Biomedical Sciences and Engineering, National Central University, Taoyuan City 320317, Taiwan R.O.C.
[2] Faculty of Applied Sciences, Ton Duc Thang University, Ho Chi Minh City 700000, Vietnam
[3] Department of Chemical and Materials Engineering, National Central University, Taoyuan City 320317, Taiwan R.O.C.
* Correspondence: Department of Biomedical Sciences and Engineering, National Central University. No. 300 Jhongda Rd., Taoyuan City 320317, Taiwan R.O.C. Telephone: (+886)-3-422-7151 Ext# 27755; Fax: (+886)-3-425-3427; E-mail: yuhsianl@ncu.edu.tw

■ Supplementary Materials

S1. Result of 16sRNA sequence of *L. plantarum* MA

GTCCATGCGGCGTGCTATACGTGCAAGTCGAACGAACTCTGGTATTGATTGGTGCT
TGCATCATGATTTACATTTGAGTGAGTGGCGAACTGGTGAGTAACACGTGGGAAAC
CTGCCCAGAAGCGGGGGATAACACCTGGAAACAGATGCTAATACCGCATAACAAC
TTGGACCGCATGGTCCGAGTTTGAAAGATGGCTTCGGCTATCACTTTTGGATGGTC
CCGCGGCGTATTAGCTAGATGGTGGGGTAACGGCTCACCATGGCAATGATACGTAG
CCGACCTGAGAGGGTAATCGGCCACATTGGGACTGAGACACGGCCCAAACTCCTA
CGGGAGGCAGCAGTAGGGAATCTTCCACAATGGACGAAAGTCTGATGGAGCAAC
GCCGCGTGAGTGAAGAAGGGTTTCGGCTCGTAAAACTCTGTTGTTAAAGAAGAAC
ATATCTGAGAGTAACTGTTCAGGTATTGACGGTATTTAACCAGAAAGCCACGGCTA
ACTACGTGCCAGCAGCGGCGGTAATGA
